# Kallolide A acetate pyrazoline

**DOI:** 10.1107/S1600536811051890

**Published:** 2011-12-07

**Authors:** Idaliz Rodríguez-Escudero, Jeffrey Marrero, Abimael D. Rodríguez

**Affiliations:** a PO Box 70377, University of Puerto Rico, San Juan, PR 00936-0377, Puerto Rico

## Abstract

In the crystal structure of kallolide A acetate pyrazoline [systematic name: 7-methyl-16-oxo-4,10-bis­(prop-1-en-2-yl)-17,18-dioxa-14,15-diaza­tetra­cyclo­[9.4.2.1^6,9^.0^1,12^]octa­deca-6,8,14-trien-5-yl acetate], C_23_H_28_N_2_O_5_, there is a 12-member­ed carbon macrocyclic structure. In addition, there is a tris­ubstituted furan ring, an approximately planar γ-lactone ring [maximum deviation of 0.057 (3) Å] and a pyraz­oline ring, the latter in an envelope conformation. The pyrazoline and the γ-lactone rings are fused in a *cis* configuration. In the crystal, mol­ecules are linked by weak C—H⋯O inter­actions, forming a two-dimensional network parallel to (001). An intra­molecular C—H⋯O hydrogen bond is also present.

## Related literature

For information on West Indies sea plumes, see: Bayer (1961 ▶); Lasker & Coffroth (1983 ▶); Humman (1996 ▶); Sánchez *et al.* (1998 ▶); Williams & Vennam (2001 ▶). For complete background to the natural product chemistry of the Gorgonian genus *Pseudopterogorgia*, see: Marrero *et al.* (2010 ▶). For species of *Pseudopterogorgia*, see: Yoshioka (1997 ▶); Sánchez *et al.* (2003 ▶); Sánchez & Lasker (2003 ▶). For the biological activity of diterpenoids from *Pseudopterogorgia*, see: Heckrodt & Mulzer (2005 ▶). For more information on the pseudoterane-type of diterpenes, see: Bundurraga & Fenical (1982 ▶); Look *et al.* (1985 ▶); Williams *et al.* (1987*b* ▶); Rodríguez & Soto (1996 ▶); Marrero *et al.* (2006 ▶). For bioactive diterpenes isolated from *Pseudopterogorgia kallos*, see: Marrero *et al.* (2003*a* ▶,*b* ▶, 2004*a* ▶,*b* ▶, 2005 ▶). For biosynthetic relationship studies between cembrane- and pseudopterane-type diterpenes, see: Rodríguez & Shi (1998 ▶); Yang *et al.* (2010 ▶); Li & Pattenden (2011 ▶). For information on gersolane-type diterpenes and biosynthetic relationship studies between cembrane- and gersolane-type diterpenes, see: Williams *et al.* (1987*a* ▶); Rodríguez *et al.* (1998 ▶). For complete background to the chemistry of furan­ocembranoids, pseudopteranes, gersolanes and related compounds, see: Roethle & Trauner (2008 ▶). For the synthesis of kallolide A and kallolide A acetate, see: Marshall & Liao (1998 ▶).
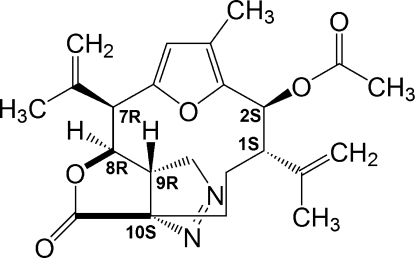


## Experimental

### 

#### Crystal data

C_23_H_28_N_2_O_5_
*M* *_r_* = 412.47
Orthorhombic, 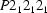

*a* = 10.593 (6) Å
*b* = 12.426 (7) Å
*c* = 17.099 (10) Å
*V* = 2251 (2) Å^3^
*Z* = 4
Mo *K*α radiation
μ = 0.09 mm^−1^
*T* = 298 K
0.40 × 0.30 × 0.10 mm

#### Data collection

Bruker SMART 1K CCD diffractometer
Absorption correction: multi-scan (*SADABS*; Sheldrick, 2008*a* ▶) *T*_min_ = 0.966, *T*_max_ = 0.992
14038 measured reflections
2583 independent reflections
2160 reflections with *I* > 2σ(*I*)
*R*_int_ = 0.041

#### Refinement

*R*[*F* ^2^ > 2σ(*F* ^2^)] = 0.047
*wR*(*F* ^2^) = 0.118
*S* = 1.13
2583 reflections
276 parameters
H-atom parameters constrained
Δρ_max_ = 0.35 e Å^−3^
Δρ_min_ = −0.23 e Å^−3^

### 

Data collection: *SMART-NT* (Bruker, 1998 ▶); cell refinement: *SAINT* (Bruker, 1999 ▶); data reduction: *SAINT*; program(s) used to solve structure: *SHELXS97* (Sheldrick, 2008*b* ▶); program(s) used to refine structure: *SHELXL97* (Sheldrick, 2008*b* ▶); molecular graphics: *SHELXTL* (Sheldrick, 2008*b* ▶); software used to prepare material for publication: *SHELXTL*.

## Supplementary Material

Crystal structure: contains datablock(s) I, global. DOI: 10.1107/S1600536811051890/wn2456sup1.cif

Supplementary material file. DOI: 10.1107/S1600536811051890/wn2456Isup2.cdx

Structure factors: contains datablock(s) I. DOI: 10.1107/S1600536811051890/wn2456Isup3.hkl

Supplementary material file. DOI: 10.1107/S1600536811051890/wn2456Isup4.cml

Additional supplementary materials:  crystallographic information; 3D view; checkCIF report

## Figures and Tables

**Table 1:** Hydrogen-bond geometry (Å, °)

*D*—H⋯*A*	*D*—H	H⋯*A*	*D*⋯*A*	*D*—H⋯*A*
C8—H8⋯O5^i^	0.98	2.37	3.281 (4)	154
C9—H9⋯O4^ii^	0.98	2.52	3.319 (4)	139
C16—H16*A*⋯O5	0.96	2.54	3.347 (5)	142

Symmetry codes: (i) 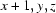; (ii) 
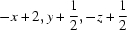
.

**Fig. 1.**
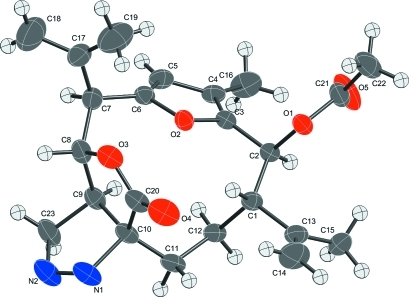
Molecular structure of the title compound. Displacement ellipsoids are shown at the 30% probability level. Hydrogen atoms are shown as spheres of arbitrary radius.

**Fig. 2.**
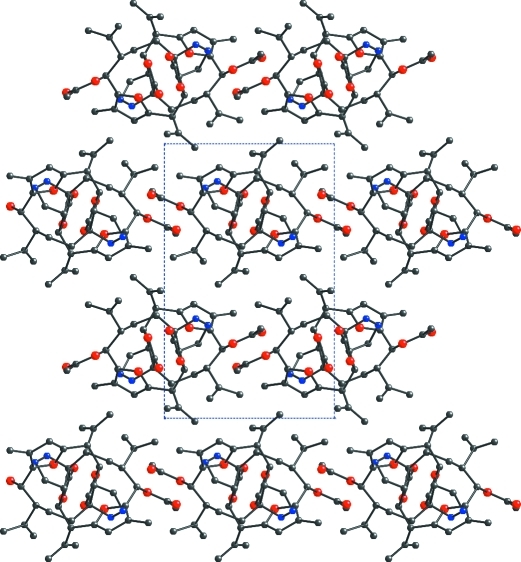
Packing view along the b axis.

**Fig. 3.**
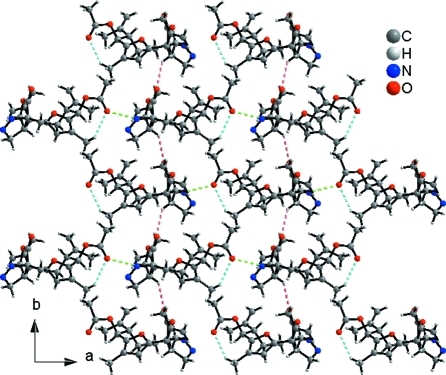
Packing view along the c axis showing the two-dimensional network formed by C—H···O intermolecular hydrogen bonding. Intramolecular C—H···O hydrogen bonds are also shown in blue.

## supplementary crystallographic information

### Comment

The title compound, in its enantiopure form, was prepared from the known pseudopterane diterpene kallolide A acetate, which was isolated from the marine sea plume *Pseudoptereogorgia* kallos.

The gorgonian octocorals of the genus *Pseudopterogorgia* are common inhabitants of tropical West Indies (Humman, 1996) and Indo-Pacific reefs. They can be adapted to different marine reef environments, from shallow to clear deep waters. Twenty two species and subspecies of *Pseudopterogorgia* have been reported and are commonly known as sea plumes for the feather-like appearance of their branches and ramifications (Bayer, 1961; Marrero *et al.*, 2010). Despite these general morphological similarities each species can be identified by differences in color, branch ramification, polyps size, texture, growth form, mucus production, sclerites, spicule, and geographical distribution (Yoshioka, 1997; Sánchez *et al.*, 2003; Sánchez & Lasker, 2003). In the West Indies region, sea plumes from this genus are commonly found from Bermuda to the Bahamas, the Florida Keys, the Greater and Lesser Antilles, and the northern coast of South America to Brazil (Bayer, 1961; Williams & Vennam, 2001; Lasker & Coffroth, 1983; Sánchez *et al.*, 1998). West Indies *Pseudopterogorgia* species are well known for the production of a variety of diterpenoids of fascinating molecular structures (Marrero *et al.*, 2010) that exhibit a wide spectrum of biological activities including antibacterial, anti-inflammatory, antimalarial, and cytotoxic properties (Heckrodt & Mulzer, 2005). An early investigation on the chemical composition of *Pseudopterogorgia* kallos showed that it is a rich source of pseudopterane-type diterpenoids (Look *et al.*, 1985). However, during the last eight years (2003–2011) subsequent chemical scrutiny has demonstrated that this gorgonian species also contains a number of minor bioactive diterpenes that are based on distinctively novel carbon frameworks (*i.e.*, bielschowskysin, ciereszkolide, intricarene, kallosin A, and providencin) (Marrero *et al.*, 2003*a*; Marrero *et al.*, 2003*b*; Marrero *et al.*, 2004*a*; Marrero *et al.*, 2004*b*; Marrero *et al.*, 2005).

The molecular structure of kallolide A acetate pyrazoline is shown in Fig. 1. It has a twelve carbon-membered macrocyclic structure with three additional rings: a trisubstituted furan, an approximately planar γ-lactone ring twisted on the C9—C10 bond, and a pyrazoline ring in an envelope conformation with C9 as the flap atom. Fused in a *cis* configuration, the angle between the mean planes of the pyrazoline and the γ-lactone rings is 111.5 (1)°.

In the crystal structure (Fig. 3), molecules are linked *via* C8— H8···O5 and C9— H9···O4 hydrogen bonds, forming a two-dimensional network. An additional intramolecular C16— H16A···O5 hydrogen bond is also present. The absolute structure was assigned as (1*S*, 2S, 7*R*, 8*R*, 9*R*, 10S), based on previous asymmetric synthesis of kallolide A and kallolide A acetate (Marshall & Liao,1998).

### Experimental

Fresh specimens of the sea plume *Pseudopterogorgia kallos* were collected by hand using SCUBA at depths of 83–91 ft in Old Providence Island, Colombia, on March 15–16, 2002. The taxonomic identification of these gorgonian species was conducted by Dr. Juan A. Sánchez (Universidad de Los Andes, Bogotá). A voucher specimen is stored in the Chemistry Department of the University of Puerto Rico, Río Piedras Campus. The organism was partially air-dried, frozen, and lyophilized prior to its extraction. The dry specimens (1.07 kg) were blended using a mixture of CH_2_Cl_2_/MeOH (1:1) (20 *x* 1 *L*). After filtration, the crude extract was concentrated and stored under vacuum to yield a greenish gum (166 g). The crude extract was suspended in water (2 *L*) and extracted with *n*-hexane (3 *x* 2 *L*), CHCl_3_ (3 *x* 2 *L*), and EtOAc (2 *x* 2 *L*). Each extract was concentrated under reduced pressure to yield 71.9 g of the *n*-hexane extract (PkH), 39.3 g of the CHCl_3_ extract (PkC), and 1.47 g of the EtOAc extract (PkA). The isolation and purification of the starting material, kallolide A acetate, for the synthesis of the title compound was achieved *via* published procedures [Marrero *et al.* (2006)]. A CHCl_3_ solution of kallolide A acetate (15 mg) was treated with an excess of CH_2_N_2_ ether solution and stirred at room temperature. After 36 h the reaction mixture was concentrated *in vacuo* to remove the ether solution of CH_2_N_2_ to yield 16.7 mg of kallolide A acetate pyrazoline as a pure colorless solid (16.7 mg, 100% yield). The title compound was recrystallized by slow evaporation using hot acetone as a solvent.

### Refinement

H atoms were positioned geometrically, with C—H = 0.96 (CH_3_), 0.97 (CH_2_), 0.98 (methine CH) and 0.93 (aromatic CH) Å, and constrained with *U*_iso_(H) = 1.5 *U*_eq_(parent) for methyl H and *U*_iso_(H) = 1.2 *U*_eq_(parent) for all other H atoms. In the absence of strong anomalous scattering, Friedel pairs were merged prior to final refinement.

### Crystal data

**Table:**

C_23_H_28_N_2_O_5_	*F*(000) = 880
*M**_r_* = 412.47	*D*_x_ = 1.217 Mg m^−^^3^
Orthorhombic, *P*2_1_2_1_2_1_	Mo *K*α radiation, λ = 0.71073 Å
Hall symbol: P 2ac 2ab	Cell parameters from 9306 reflections
*a* = 10.593 (6) Å	θ = 2.3–26.7°
*b* = 12.426 (7) Å	µ = 0.09 mm^−^^1^
*c* = 17.099 (10) Å	*T* = 298 K
*V* = 2251 (2) Å^3^	Block, colourless
*Z* = 4	0.40 × 0.30 × 0.10 mm

### Data collection

**Table:**

Bruker SMART 1K CCD diffractometer	2583 independent reflections
Radiation source: fine-focus sealed tube	2160 reflections with *I* > 2σ(*I*)
graphite	*R*_int_ = 0.041
φ and ω scans	θ_max_ = 26.4°, θ_min_ = 2.0°
Absorption correction: multi-scan (*SADABS*; Sheldrick, 2008*a*)	*h* = −11→13
*T*_min_ = 0.966, *T*_max_ = 0.992	*k* = −15→15
14038 measured reflections	*l* = −21→21

### Refinement

**Table:**

Refinement on *F*^2^	Secondary atom site location: difference Fourier map
Least-squares matrix: full	Hydrogen site location: inferred from neighbouring sites
*R*[*F*^2^ > 2σ(*F*^2^)] = 0.047	H-atom parameters constrained
*wR*(*F*^2^) = 0.118	*w* = 1/[σ^2^(*F*_o_^2^) + (0.0552*P*)^2^ + 0.3145*P*] where *P* = (*F*_o_^2^ + 2*F*_c_^2^)/3
*S* = 1.13	(Δ/σ)_max_ < 0.001
2583 reflections	Δρ_max_ = 0.35 e Å^−^^3^
276 parameters	Δρ_min_ = −0.23 e Å^−^^3^
0 restraints	Extinction correction: *SHELXL97* (Sheldrick, 2008a), Fc^*^=kFc[1+0.001xFc^2^λ^3^/sin(2θ)]^-1/4^
Primary atom site location: structure-invariant direct methods	Extinction coefficient: 0.061 (4)

### Special details

**Table:**

Experimental. IR(neat) ν_max_ 3078, 2969, 2944, 2927, 1764, 1728, 1642, 1375, 1251, 1227, 907, 810 cm^-1^; ^1^H NMR (300 MHz, DMSO-d6) δ 3.10 (1*H*, dd, *J* = 11.0, 9.0 Hz, H-2),5.60 (1*H*, d, *J* = 11.7 Hz, H-2), 6.23 (1*H*, s, H-5), 3.89 (1*H*, d, *J* = 2.7 Hz, H-7), 4.59 (1*H*, m, H-8)*^a^*, 2.11 (1*H*, br dd, *J* = 6.3, 5.7 Hz, H-9), 2.48 (1*H*, m, H-11*a*)*^b^*, 0.81 (1*H*, dd, *J* = 14.1, 5.7 Hz), 1.31 (1*H*,m,H-12*a*), 0.30 (1*H*, dd, *J* = 13.8, 13.5 Hz, H-12*b*), 4.88 (1*H*,vd, *J* = 1.2 Hz, H-14*a*), 4.51 (1*H*, s, H-14*b*), 1.77 (3*H*, s, H-15), 2.00 (3*H*, s, H-16), 5.08 (1*H*, d, *J* = 2.1 Hz, H-18*a*), 4.93 (1*H*, s, H-18*b*), 1.63 (3*H*, s, H-19), 1.94 (3*H*, s, H-22), 5.17 (1*H*, d, *J* = 18.6 Hz, H-23*a*), 5.62 (1*H*, dd, *J* = 18.9 Hz, H-23*b*)*^a^* (*^a^* values are interchangeable, *^b^* proton signal peak overlap with solvent). ^13^C NMR (DMSO-d6, 75 MHz) δ 48.4 (CH, C-1), 66.5 (CH, C-2), 146.5 (C, C-3), 122.4 (C, C4), 114.4 (CH, C-5), 152.5 (C, C-6), 46.0 (CH, C-7), 84.6 (CH, C-8), 36.7 (CH, C-9), 105.7 (C, C-10), 26.6 (CH_2_, C-11), 25.1 (CH_2_, C-12), 143.9 (C, C-13), 114.4 (CH_2_, C-14), 21.8 (CH_3_, C-15), 9.7 (CH_3_, C-16), 143.3 (C, C-17), 115.5 (CH_2_, C-18), 17.9 (CH_3_, C-19), 172.9 (C, C-10), 170.1 (C, C-21), 21.1 (CH_3_, C-22), 86.2 (CH_2_, C-23); LREI-MS m/*z* [*M*]^+^; 412(6.4), 384 (19), 370 (15), 342 (9), 231 (7), 214 (13), 178 (11), 165 (23), 164 (100), 163 (87), 135 (28); HREI-MS *m/z* [*M*]^+^ calcd for C_23_H_28_N_2_O_5_ 412.1998 found 412.2003.
Geometry. All e.s.d.'s (except the e.s.d. in the dihedral angle between two l.s. planes) are estimated using the full covariance matrix. The cell e.s.d.'s are taken into account individually in the estimation of e.s.d.'s in distances, angles and torsion angles; correlations between e.s.d.'s in cell parameters are only used when they are defined by crystal symmetry. An approximate (isotropic) treatment of cell e.s.d.'s is used for estimating e.s.d.'s involving l.s. planes.
Refinement. Refinement of *F*^2^ against ALL reflections. The weighted *R*-factor *wR* and goodness of fit *S* are based on *F*^2^, conventional *R*-factors *R* are based on *F*, with *F* set to zero for negative *F*^2^. The threshold expression of *F*^2^ > σ(*F*^2^) is used only for calculating *R*-factors(gt) *etc*. and is not relevant to the choice of reflections for refinement. *R*-factors based on *F*^2^ are statistically about twice as large as those based on *F*, and *R*- factors based on ALL data will be even larger.

### Fractional atomic coordinates and isotropic or equivalent isotropic displacement parameters (Å^2^)

**Table:**

	*x*	*y*	*z*	*U*_iso_*/*U*_eq_	
O2	0.85009 (16)	0.35559 (14)	0.16945 (10)	0.0506 (4)	
O1	0.59698 (18)	0.25015 (14)	0.22854 (11)	0.0564 (5)	
C3	0.7288 (2)	0.39670 (19)	0.17940 (15)	0.0474 (6)	
C9	1.0882 (3)	0.4597 (2)	0.24459 (17)	0.0586 (7)	
H9	1.0151	0.5047	0.2316	0.070*	
O5	0.4266 (2)	0.3406 (2)	0.1863 (2)	0.1156 (11)	
O3	1.0951 (2)	0.27160 (17)	0.20733 (13)	0.0696 (6)	
C20	1.0579 (3)	0.2727 (2)	0.28330 (18)	0.0629 (8)	
C11	0.9562 (3)	0.4146 (2)	0.37422 (16)	0.0629 (8)	
H11A	0.9813	0.4790	0.4023	0.076*	
H11B	0.9513	0.3568	0.4122	0.076*	
O4	1.0311 (3)	0.19117 (16)	0.31793 (15)	0.0867 (8)	
C6	0.9085 (3)	0.4155 (2)	0.11168 (14)	0.0526 (6)	
C2	0.6586 (3)	0.35346 (19)	0.24830 (14)	0.0486 (6)	
H2	0.5943	0.4056	0.2645	0.058*	
C1	0.7473 (3)	0.3298 (2)	0.31789 (14)	0.0517 (6)	
H1	0.8090	0.2763	0.3003	0.062*	
C10	1.0619 (3)	0.3868 (2)	0.31621 (17)	0.0576 (7)	
N2	1.2578 (3)	0.4666 (3)	0.3389 (2)	0.0925 (10)	
C12	0.8225 (3)	0.4334 (2)	0.34163 (15)	0.0553 (7)	
H12A	0.8291	0.4796	0.2961	0.066*	
H12B	0.7739	0.4720	0.3807	0.066*	
C4	0.7090 (3)	0.4796 (2)	0.12735 (14)	0.0555 (7)	
C5	0.8259 (3)	0.4905 (2)	0.08522 (15)	0.0620 (8)	
H5	0.8421	0.5407	0.0462	0.074*	
C8	1.1207 (3)	0.3803 (3)	0.17747 (19)	0.0647 (8)	
H8	1.2113	0.3859	0.1667	0.078*	
N1	1.1882 (3)	0.3930 (2)	0.36229 (18)	0.0823 (9)	
C7	1.0488 (3)	0.3979 (3)	0.09993 (17)	0.0652 (8)	
H7	1.0808	0.4662	0.0792	0.078*	
C23	1.2011 (3)	0.5276 (3)	0.2723 (2)	0.0832 (10)	
H23A	1.2621	0.5365	0.2305	0.100*	
H23B	1.1733	0.5982	0.2894	0.100*	
C21	0.4806 (3)	0.2569 (3)	0.19643 (19)	0.0699 (8)	
C13	0.6784 (3)	0.2824 (2)	0.38899 (16)	0.0685 (9)	
C16	0.5932 (4)	0.5490 (3)	0.1176 (2)	0.0900 (12)	
H16A	0.5250	0.5194	0.1477	0.135*	
H16B	0.5697	0.5511	0.0634	0.135*	
H16C	0.6111	0.6206	0.1354	0.135*	
C15	0.5551 (4)	0.3321 (3)	0.41338 (19)	0.0872 (11)	
H15A	0.4911	0.3143	0.3758	0.131*	
H15B	0.5643	0.4089	0.4161	0.131*	
H15C	0.5312	0.3049	0.4638	0.131*	
C22	0.4304 (4)	0.1483 (3)	0.1731 (2)	0.0971 (13)	
H22A	0.3673	0.1257	0.2099	0.146*	
H22B	0.4982	0.0970	0.1725	0.146*	
H22C	0.3936	0.1528	0.1219	0.146*	
C17	1.0837 (4)	0.3138 (4)	0.03873 (19)	0.0848 (11)	
C19	1.0205 (7)	0.2132 (4)	0.0357 (3)	0.135 (2)	
H19A	1.0489	0.1736	−0.0092	0.202*	
H19B	0.9311	0.2249	0.0319	0.202*	
H19C	1.0387	0.1730	0.0823	0.202*	
C14	0.7312 (5)	0.2005 (3)	0.4285 (2)	0.1048 (14)	
H14A	0.6914	0.1727	0.4725	0.126*	
H14B	0.8076	0.1716	0.4118	0.126*	
C18	1.1806 (6)	0.3379 (6)	−0.0128 (3)	0.191 (3)	
H18A	1.2052	0.2878	−0.0502	0.229*	
H18B	1.2210	0.4043	−0.0100	0.229*	

### Atomic displacement parameters (Å^2^)

**Table:**

	*U*^11^	*U*^22^	*U*^33^	*U*^12^	*U*^13^	*U*^23^
O2	0.0472 (9)	0.0503 (9)	0.0543 (10)	0.0012 (8)	−0.0055 (8)	0.0105 (8)
O1	0.0550 (11)	0.0550 (10)	0.0590 (10)	−0.0065 (9)	−0.0097 (9)	0.0015 (8)
C3	0.0486 (13)	0.0441 (12)	0.0495 (13)	0.0021 (11)	−0.0095 (11)	−0.0005 (11)
C9	0.0533 (15)	0.0518 (14)	0.0706 (16)	−0.0075 (13)	−0.0157 (14)	0.0067 (13)
O5	0.0589 (14)	0.0964 (19)	0.192 (3)	0.0018 (14)	−0.0418 (19)	−0.001 (2)
O3	0.0711 (13)	0.0584 (12)	0.0792 (14)	0.0083 (11)	−0.0173 (12)	−0.0034 (10)
C20	0.0616 (18)	0.0524 (16)	0.0748 (19)	0.0048 (14)	−0.0254 (15)	0.0057 (14)
C11	0.083 (2)	0.0551 (16)	0.0502 (14)	−0.0120 (15)	−0.0210 (14)	0.0033 (13)
O4	0.1109 (19)	0.0470 (11)	0.1021 (16)	−0.0006 (12)	−0.0299 (15)	0.0179 (11)
C6	0.0571 (15)	0.0547 (14)	0.0460 (13)	−0.0065 (13)	−0.0059 (12)	0.0063 (12)
C2	0.0500 (13)	0.0472 (12)	0.0488 (13)	−0.0001 (12)	−0.0041 (11)	−0.0012 (11)
C1	0.0610 (15)	0.0471 (13)	0.0469 (13)	−0.0039 (12)	−0.0069 (12)	0.0030 (11)
C10	0.0609 (16)	0.0500 (14)	0.0620 (16)	−0.0036 (12)	−0.0251 (14)	0.0059 (12)
N2	0.078 (2)	0.090 (2)	0.109 (2)	−0.0219 (18)	−0.0372 (18)	0.0025 (18)
C12	0.0708 (18)	0.0495 (14)	0.0457 (12)	−0.0050 (13)	−0.0063 (13)	0.0004 (11)
C4	0.0660 (17)	0.0563 (14)	0.0443 (12)	0.0099 (14)	−0.0104 (12)	0.0017 (11)
C5	0.081 (2)	0.0597 (16)	0.0455 (13)	−0.0025 (16)	−0.0075 (14)	0.0129 (12)
C8	0.0509 (15)	0.0694 (18)	0.0738 (19)	−0.0056 (14)	−0.0058 (14)	0.0060 (15)
N1	0.0778 (19)	0.0810 (18)	0.0882 (19)	−0.0025 (16)	−0.0410 (16)	0.0061 (16)
C7	0.0586 (17)	0.0775 (19)	0.0594 (16)	−0.0087 (15)	−0.0001 (13)	0.0094 (15)
C23	0.076 (2)	0.0747 (19)	0.099 (2)	−0.0248 (19)	−0.024 (2)	0.0073 (19)
C21	0.0514 (16)	0.082 (2)	0.0764 (19)	−0.0127 (16)	−0.0093 (15)	0.0080 (17)
C13	0.086 (2)	0.0729 (18)	0.0464 (14)	−0.0235 (18)	−0.0050 (15)	0.0042 (14)
C16	0.104 (3)	0.089 (2)	0.077 (2)	0.043 (2)	−0.007 (2)	0.0167 (19)
C15	0.094 (3)	0.106 (3)	0.0609 (18)	−0.026 (2)	0.0143 (18)	−0.0068 (18)
C22	0.092 (3)	0.099 (3)	0.101 (3)	−0.040 (2)	−0.030 (2)	0.010 (2)
C17	0.069 (2)	0.123 (3)	0.0621 (18)	0.005 (2)	0.0067 (17)	−0.0059 (19)
C19	0.178 (6)	0.116 (4)	0.110 (3)	−0.003 (4)	0.035 (4)	−0.032 (3)
C14	0.131 (4)	0.107 (3)	0.076 (2)	−0.023 (3)	−0.009 (2)	0.041 (2)
C18	0.154 (5)	0.275 (8)	0.143 (5)	−0.080 (6)	0.086 (4)	−0.082 (5)

### Geometric parameters (Å, °)

**Table:**

O2—C6	1.383 (3)	C4—C5	1.439 (4)
O2—C3	1.393 (3)	C4—C16	1.509 (4)
O1—C21	1.353 (4)	C5—H5	0.9300
O1—C2	1.479 (3)	C8—C7	1.545 (4)
C3—C4	1.378 (4)	C8—H8	0.9800
C3—C2	1.493 (4)	C7—C17	1.524 (5)
C9—C23	1.539 (4)	C7—H7	0.9800
C9—C10	1.549 (4)	C23—H23A	0.9700
C9—C8	1.552 (4)	C23—H23B	0.9700
C9—H9	0.9800	C21—C22	1.505 (5)
O5—C21	1.200 (4)	C13—C14	1.343 (5)
O3—C20	1.358 (4)	C13—C15	1.504 (5)
O3—C8	1.470 (4)	C16—H16A	0.9600
C20—O4	1.207 (4)	C16—H16B	0.9600
C20—C10	1.526 (4)	C16—H16C	0.9600
C11—C10	1.535 (5)	C15—H15A	0.9600
C11—C12	1.540 (4)	C15—H15B	0.9600
C11—H11A	0.9700	C15—H15C	0.9600
C11—H11B	0.9700	C22—H22A	0.9600
C6—C5	1.356 (4)	C22—H22B	0.9600
C6—C7	1.516 (4)	C22—H22C	0.9600
C2—C1	1.544 (3)	C17—C18	1.386 (6)
C2—H2	0.9800	C17—C19	1.419 (6)
C1—C13	1.536 (4)	C19—H19A	0.9600
C1—C12	1.567 (4)	C19—H19B	0.9600
C1—H1	0.9800	C19—H19C	0.9600
C10—N1	1.555 (4)	C14—H14A	0.9300
N2—N1	1.241 (4)	C14—H14B	0.9300
N2—C23	1.493 (5)	C18—H18A	0.9300
C12—H12A	0.9700	C18—H18B	0.9300
C12—H12B	0.9700		
			
C6—O2—C3	107.6 (2)	C7—C8—C9	115.8 (2)
C21—O1—C2	116.2 (2)	O3—C8—H8	108.2
C4—C3—O2	109.6 (2)	C7—C8—H8	108.2
C4—C3—C2	134.7 (2)	C9—C8—H8	108.2
O2—C3—C2	115.1 (2)	N2—N1—C10	112.6 (3)
C23—C9—C10	102.5 (2)	C6—C7—C17	115.3 (3)
C23—C9—C8	113.8 (3)	C6—C7—C8	113.0 (2)
C10—C9—C8	104.7 (2)	C17—C7—C8	111.9 (3)
C23—C9—H9	111.7	C6—C7—H7	105.2
C10—C9—H9	111.7	C17—C7—H7	105.2
C8—C9—H9	111.7	C8—C7—H7	105.2
C20—O3—C8	112.1 (2)	N2—C23—C9	105.6 (2)
O4—C20—O3	122.0 (3)	N2—C23—H23A	110.6
O4—C20—C10	127.3 (3)	C9—C23—H23A	110.6
O3—C20—C10	110.7 (3)	N2—C23—H23B	110.6
C10—C11—C12	118.1 (2)	C9—C23—H23B	110.6
C10—C11—H11A	107.8	H23A—C23—H23B	108.8
C12—C11—H11A	107.8	O5—C21—O1	123.1 (3)
C10—C11—H11B	107.8	O5—C21—C22	124.9 (3)
C12—C11—H11B	107.8	O1—C21—C22	112.0 (3)
H11A—C11—H11B	107.1	C14—C13—C15	122.3 (3)
C5—C6—O2	108.6 (2)	C14—C13—C1	119.4 (3)
C5—C6—C7	133.5 (3)	C15—C13—C1	118.3 (3)
O2—C6—C7	117.1 (2)	C4—C16—H16A	109.5
O1—C2—C3	110.6 (2)	C4—C16—H16B	109.5
O1—C2—C1	106.24 (18)	H16A—C16—H16B	109.5
C3—C2—C1	111.9 (2)	C4—C16—H16C	109.5
O1—C2—H2	109.3	H16A—C16—H16C	109.5
C3—C2—H2	109.3	H16B—C16—H16C	109.5
C1—C2—H2	109.3	C13—C15—H15A	109.5
C13—C1—C2	113.2 (2)	C13—C15—H15B	109.5
C13—C1—C12	110.6 (2)	H15A—C15—H15B	109.5
C2—C1—C12	110.7 (2)	C13—C15—H15C	109.5
C13—C1—H1	107.4	H15A—C15—H15C	109.5
C2—C1—H1	107.4	H15B—C15—H15C	109.5
C12—C1—H1	107.4	C21—C22—H22A	109.5
C20—C10—C11	115.3 (3)	C21—C22—H22B	109.5
C20—C10—C9	104.9 (2)	H22A—C22—H22B	109.5
C11—C10—C9	120.7 (2)	C21—C22—H22C	109.5
C20—C10—N1	104.9 (2)	H22A—C22—H22C	109.5
C11—C10—N1	106.8 (2)	H22B—C22—H22C	109.5
C9—C10—N1	102.5 (2)	C18—C17—C19	121.1 (5)
N1—N2—C23	112.4 (3)	C18—C17—C7	117.9 (5)
C11—C12—C1	115.9 (2)	C19—C17—C7	121.0 (3)
C11—C12—H12A	108.3	C17—C19—H19A	109.5
C1—C12—H12A	108.3	C17—C19—H19B	109.5
C11—C12—H12B	108.3	H19A—C19—H19B	109.5
C1—C12—H12B	108.3	C17—C19—H19C	109.5
H12A—C12—H12B	107.4	H19A—C19—H19C	109.5
C3—C4—C5	105.2 (2)	H19B—C19—H19C	109.5
C3—C4—C16	128.5 (3)	C13—C14—H14A	120.0
C5—C4—C16	126.2 (3)	C13—C14—H14B	120.0
C6—C5—C4	108.9 (2)	H14A—C14—H14B	120.0
C6—C5—H5	125.5	C17—C18—H18A	120.0
C4—C5—H5	125.5	C17—C18—H18B	120.0
O3—C8—C7	109.7 (2)	H18A—C18—H18B	120.0
O3—C8—C9	106.6 (2)		
			
C6—O2—C3—C4	1.6 (3)	C2—C3—C4—C16	−8.9 (5)
C6—O2—C3—C2	−170.6 (2)	O2—C6—C5—C4	0.2 (3)
C8—O3—C20—O4	179.1 (3)	C7—C6—C5—C4	−168.9 (3)
C8—O3—C20—C10	−3.5 (3)	C3—C4—C5—C6	0.7 (3)
C3—O2—C6—C5	−1.1 (3)	C16—C4—C5—C6	178.4 (3)
C3—O2—C6—C7	170.1 (2)	C20—O3—C8—C7	−129.1 (3)
C21—O1—C2—C3	−87.0 (3)	C20—O3—C8—C9	−2.9 (3)
C21—O1—C2—C1	151.3 (2)	C23—C9—C8—O3	119.0 (3)
C4—C3—C2—O1	106.3 (3)	C10—C9—C8—O3	7.9 (3)
O2—C3—C2—O1	−84.0 (2)	C23—C9—C8—C7	−118.6 (3)
C4—C3—C2—C1	−135.4 (3)	C10—C9—C8—C7	130.2 (3)
O2—C3—C2—C1	34.2 (3)	C23—N2—N1—C10	−0.7 (4)
O1—C2—C1—C13	−57.6 (3)	C20—C10—N1—N2	122.8 (3)
C3—C2—C1—C13	−178.4 (2)	C11—C10—N1—N2	−114.3 (3)
O1—C2—C1—C12	177.6 (2)	C9—C10—N1—N2	13.5 (4)
C3—C2—C1—C12	56.7 (3)	C5—C6—C7—C17	−99.1 (4)
O4—C20—C10—C11	−39.2 (4)	O2—C6—C7—C17	92.5 (3)
O3—C20—C10—C11	143.7 (2)	C5—C6—C7—C8	130.4 (3)
O4—C20—C10—C9	−174.4 (3)	O2—C6—C7—C8	−38.0 (4)
O3—C20—C10—C9	8.4 (3)	O3—C8—C7—C6	74.9 (3)
O4—C20—C10—N1	78.0 (4)	C9—C8—C7—C6	−45.8 (4)
O3—C20—C10—N1	−99.2 (3)	O3—C8—C7—C17	−57.2 (3)
C12—C11—C10—C20	−74.1 (3)	C9—C8—C7—C17	−178.0 (3)
C12—C11—C10—C9	53.6 (3)	N1—N2—C23—C9	−12.6 (4)
C12—C11—C10—N1	169.9 (2)	C10—C9—C23—N2	19.6 (3)
C23—C9—C10—C20	−128.7 (3)	C8—C9—C23—N2	−92.8 (3)
C8—C9—C10—C20	−9.6 (3)	C2—O1—C21—O5	−2.4 (5)
C23—C9—C10—C11	99.1 (3)	C2—O1—C21—C22	175.7 (3)
C8—C9—C10—C11	−141.8 (2)	C2—C1—C13—C14	137.6 (3)
C23—C9—C10—N1	−19.3 (3)	C12—C1—C13—C14	−97.6 (3)
C8—C9—C10—N1	99.8 (2)	C2—C1—C13—C15	−44.3 (3)
C10—C11—C12—C1	76.3 (3)	C12—C1—C13—C15	80.6 (3)
C13—C1—C12—C11	85.8 (3)	C6—C7—C17—C18	136.5 (5)
C2—C1—C12—C11	−147.9 (2)	C8—C7—C17—C18	−92.6 (5)
O2—C3—C4—C5	−1.4 (3)	C6—C7—C17—C19	−44.7 (5)
C2—C3—C4—C5	168.6 (3)	C8—C7—C17—C19	86.2 (5)
O2—C3—C4—C16	−178.9 (3)		

### Hydrogen-bond geometry (Å, °)

**Table:**

*D*—H···*A*	*D*—H	H···*A*	*D*···*A*	*D*—H···*A*
C8—H8···O5^i^	0.98	2.37	3.281 (4)	154.
C9—H9···O4^ii^	0.98	2.52	3.319 (4)	139.
C16—H16A···O5	0.96	2.54	3.347 (5)	142.

Symmetry codes: (i) *x*+1, *y*, *z*; (ii) −*x*+2, *y*+1/2, −*z*+1/2.
